# The rise and fall of lateral electrical surface stimulation (LESS) through the lens of SRS archives

**DOI:** 10.1007/s43390-026-01510-y

**Published:** 2026-07-27

**Authors:** Ali Rezazadeh Shirazi, Jay I. Kumar, Katie Dold, George H. Thompson, Andrew G. King, Richard M. Schwend, John P. Lubicky, Jay Shapiro, Behrooz A. Akbarnia

**Affiliations:** 1 Kansas City University, Kansas City, MO USA; 2 Department of Neurosurgery, Brain & Spine, University of South Florida, Florida, USA; 3 Medical Center, University of Kansas, Kansas City, KS USA; 4 Division of Pediatric Orthopaedic Surgery, Rainbow Babies and Children’s Hospital, University Hospitals Cleveland Medical Center, Case Western Reserve University, Cleveland, OH USA; 5 LSU Health New Orleans School of Medicine, New Orleans, Louisiana, U.S.A.; 6 Children’s Mercy Hospital, Kansas City, MO USA; 7 Department of Orthopedics, West Virginia University SOM, Morgantown, WV USA; 8 Orthopedics, Dell Medical School, University of Texas at Austin, Austin, TX USA; 9 University of California, San Diego and San Diego Spine Foundation, San Diego, USA

**Keywords:** ScoliTron™, Lateral electrical surface stimulation LESS, Adolescence idiopathic scoliosis, Scoliosis research society, Non-operative scoliosis management

## Abstract

**Purpose:**

Lateral electrical surface stimulation (LESS), commercially known as the ScoliTron™, emerged in the late 1970s as a noninvasive alternative to bracing for adolescent idiopathic scoliosis. Early clinical reports generated considerable enthusiasm and adoption, but the technique was ultimately abandoned. This study examined the rise and fall of LESS through a combined analysis of the Scoliosis Research Society (SRS) archives and published literature, with the goal of understanding factors that influence the adoption, evaluation, and abandonment of innovation in scoliosis care.

**Methods:**

A structured historical review was conducted using archival materials from the Scoliosis Research Society (SRS), including meeting abstracts, audiovisual records, and correspondence. Archival data were independently screened and extracted by two reviewers. In parallel, a focused literature review was performed in accordance with PRISMA guidelines using PubMed and Ovid MEDLINE, with independent screening and full-text assessment by two reviewers.

**Results:**

The archival review identified 24 SRS abstracts related to LESS. The literature search identified 50 records, of which 26 underwent full-text review and 12 met inclusion criteria. Early reports suggested short-term curve stabilization in selected patients, leading to increased clinical adoption. However, larger studies with longer follow-up demonstrated inconsistent outcomes, including continued progression in skeletally immature patients, variable compliance, device related complications, and high failure rates. Comparative studies consistently demonstrated outcomes inferior or, at best, equivalent to bracing, with no convincing evidence that LESS altered the natural history of scoliosis. Archival materials further revealed evolving professional perspectives and concerns that were not always captured in the published literature.

**Conclusion:**

The history of LESS illustrates how promising innovations can achieve widespread acceptance before sufficient evidence is available to support their long-term effectiveness. The SRS archives provide unique insight into the scientific, clinical, and cultural factors that shaped both the adoption and eventual abandonment of this technology. Beyond the story of LESS, this analysis highlights the value of historical archives in understanding the evolution of scoliosis care and informing future innovation.

**Supplementary Information:**

The online version contains supplementary material available at https://doi.org/10.1007/s43390-026-01510-y.

## Introduction

Lateral electrical surface stimulation (LESS), commercially known as the ScoliTron™, emerged in the late 1970 s as a noninvasive alternative to bracing for the treatment of adolescent idiopathic scoliosis [1]. At a time when treatment options were largely limited to rigid bracing or surgical correction, LESS was developed based on the premise that asymmetric muscle activation could generate corrective forces on the spine and influence curve progression. Early experimental work by Axelgaard and colleagues investigated the biomechanical effects of electrical stimulation in inducing spinal curvature in cat models [2]. In these studies, medial electrode placements positioned approximately 3 cm lateral to the spinous processes produced spinal curvatures of approximately 15°, whereas lateral placements along the mid-axillary line generated significantly larger curves, reaching up to 35° in some cases. These differences were attributed to variations in mechanical leverage and spinal flexibility. Based on these findings, investigators began to consider whether lateral electrical stimulation could be applied therapeutically to reduce, rather than induce, scoliotic deformity.

**Fig. 1:**
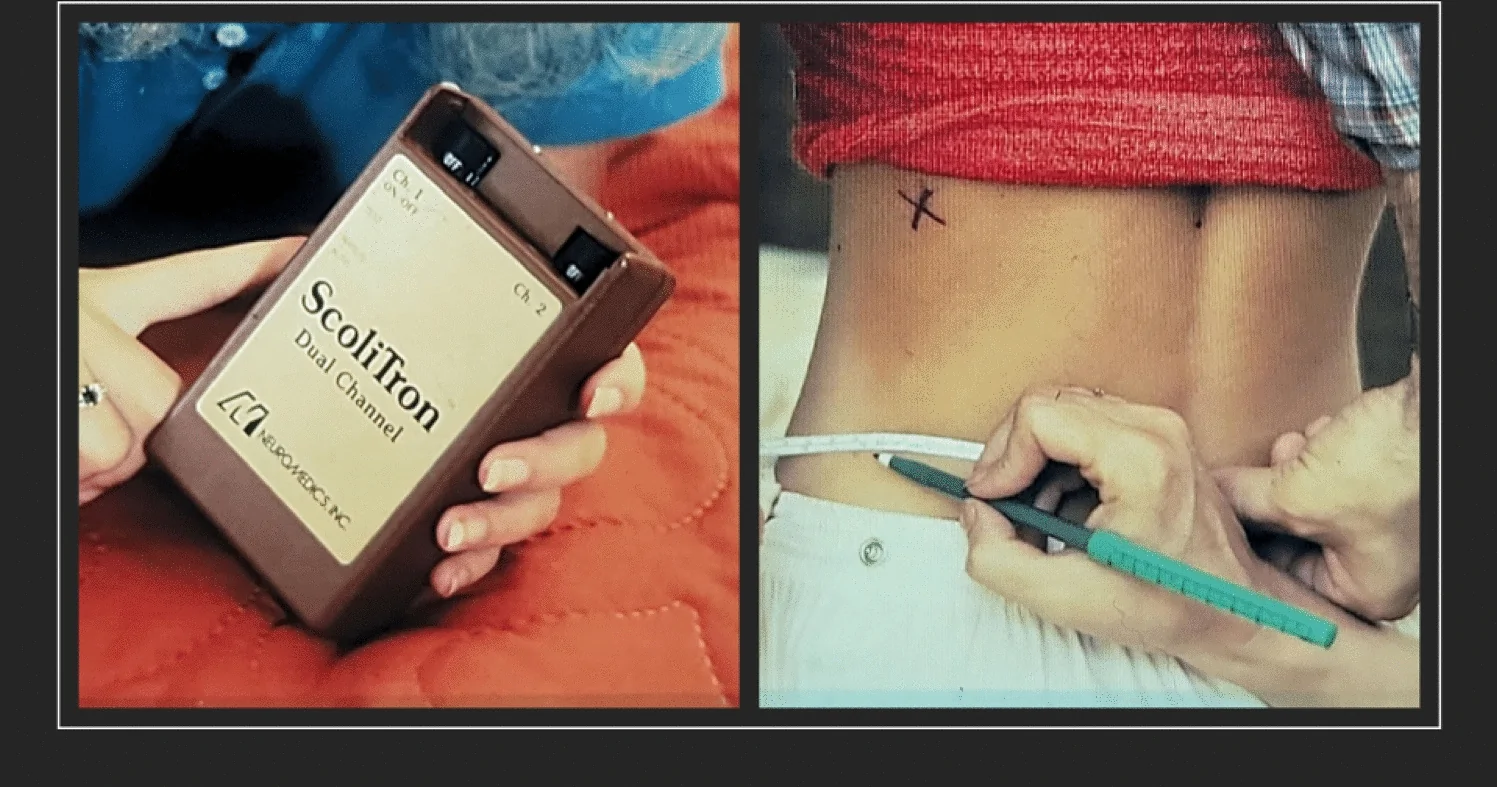
ScoliTron™ device and placement site

**Fig. 2:**
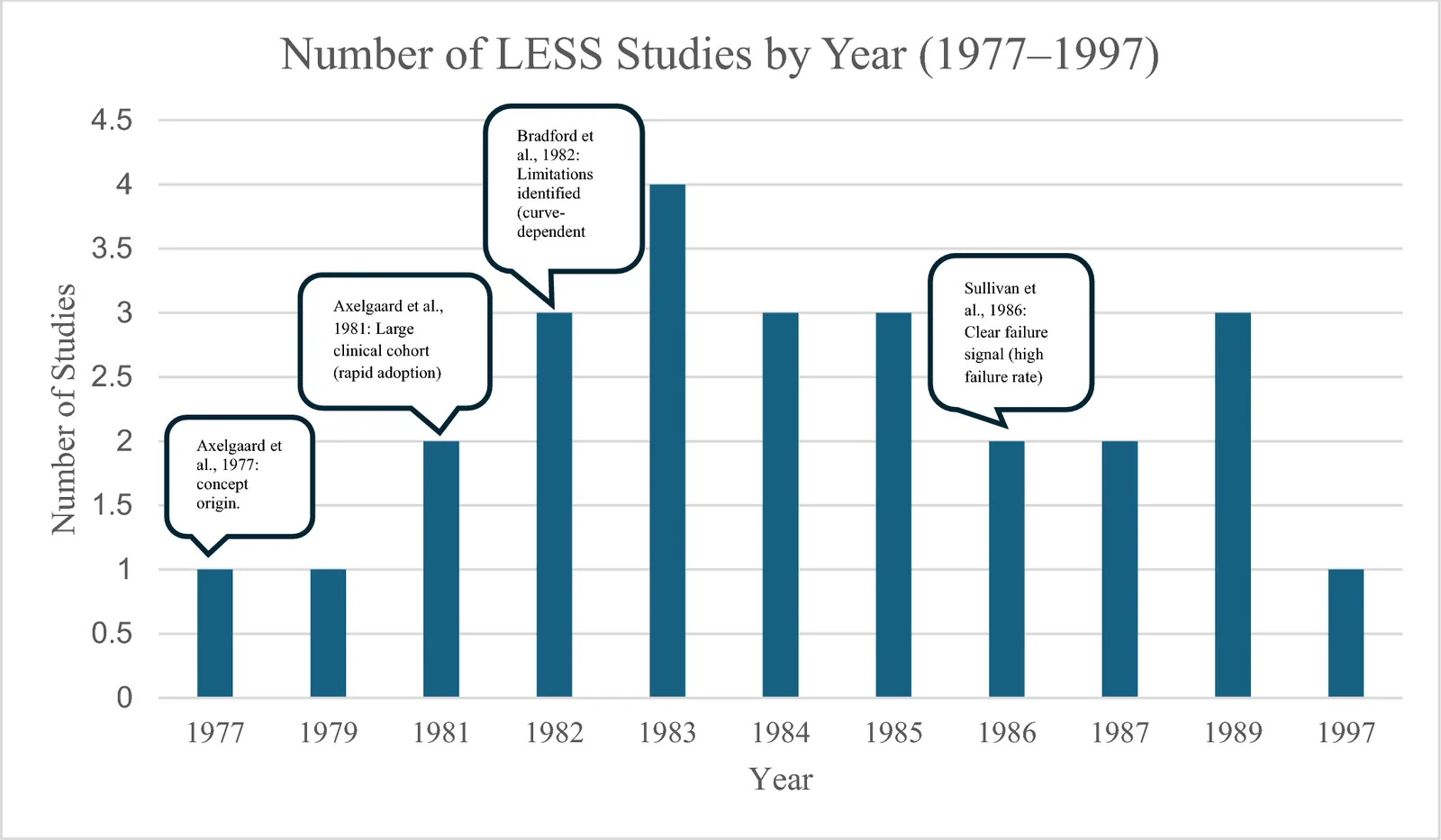
Number of studies on lateral electrical surface stimulation (LESS) for scoliosis published between 1977 and 1997, as identified in the Scoliosis Research Society archives. Key milestones in the development, clinical adoption, and decline of LESS are annotated. The peak in 1983 reflects the height of research activity, followed by a decline corresponding to increasing evidence of limited clinical effectiveness

Subsequent experimental and early clinical investigations focused on optimizing electrode placement and stimulation parameters for therapeutic use. Multiple electrode configurations were evaluated, and stimulation protocols were developed using constant-current pulses (approximately 30 pulses per second with pulse durations of 200 microseconds), with electrode placement guided by visible muscle contraction and radiographic changes [3]. The clinical application of this technique, including electrode positioning and device configuration, is illustrated in Figs. 1 and 2. These early investigations assumed that scoliosis progression could be influenced primarily through asymmetric muscle activity. Initial clinical reports suggested that lateral electrical stimulation could produce short-term curve stabilization or improvement in selected patients, generating substantial enthusiasm and leading to broader clinical adoption and multicenter investigations [4].

The development of the ScoliTron™ device represented the translation of these experimental and early clinical findings into a practical treatment modality. Designed for prolonged home use, typically during sleep, the device aimed to deliver sustained lateral muscle stimulation to influence spinal growth and curve progression [3]. However, as clinical experience expanded and larger patient cohorts were studied with longer follow-up, outcomes became increasingly inconsistent. Subsequent investigations demonstrated high failure rates, continued curve progression in skeletally immature patients, and no convincing evidence that LESS altered the natural history of scoliosis. These findings highlighted important limitations of the underlying biomechanical assumptions, which did not account for the complex three-dimensional and growth-dependent nature of scoliosis.

The Scoliosis Research Society (SRS) archives provide a unique opportunity to examine the rise, peak, and subsequent decline of LESS by capturing experimental work, early clinical experience, and evolving professional perspectives that were not always fully represented in the published literature. Through a combined analysis of archival materials and published studies, this study examines the clinical, scientific, and cultural factors that shaped the trajectory of LESS, with particular attention to the reasons underlying its initial adoption and eventual abandonment. In doing so, the history of LESS serves as a case study in the promises and pitfalls of early-stage enthusiasm, highlighting the importance of critical evaluation, long-term evidence, and historical awareness in guiding future innovation in spine care.

## Materials and methods

This study was designed as a structured historical review combined with a PRISMA-guided literature review to examine the development, clinical evaluation, and eventual decline of lateral electrical surface stimulation (LESS).

### Archival analysis

A comprehensive review of archival materials from the Scoliosis Research Society (SRS) was conducted to reconstruct the historical trajectory of LESS. Archival sources included annual meeting abstracts, meeting programs, audiovisual recordings of presentations, internal correspondence, and other historical documents preserved within the SRS archives at the University of Kansas Medical Center were reviewed. Personal collections from key contributors, including those of Dr. John Carlisle Brown and Dr. Behrooz Akbarnia, were also examined to gain insight into the development, clinical reasoning, and adoption of the technology.

Relevant archival abstracts were identified and independently reviewed by two investigators. Structured data were extracted from each abstract, including title, research institution location, study design, patient population, methods, primary results, and key conclusions or lessons learned, with discrepancies resolved by consensus. Extracted data were compiled into a structured framework to enable comparison across studies. Findings were analyzed both chronologically and thematically to identify patterns in the development, clinical adoption, and subsequent decline of LESS.

### Literature review

To complement the archival analysis, a focused literature review was conducted following PRISMA guidelines for study identification and selection. Searches were performed in PubMed and Ovid MEDLINE using combinations of the following terms: “lateral electrical surface stimulation,” “LESS,” “ScoliTron,” “electrical stimulation,” and “adolescent idiopathic scoliosis.”

**Fig. 3:**
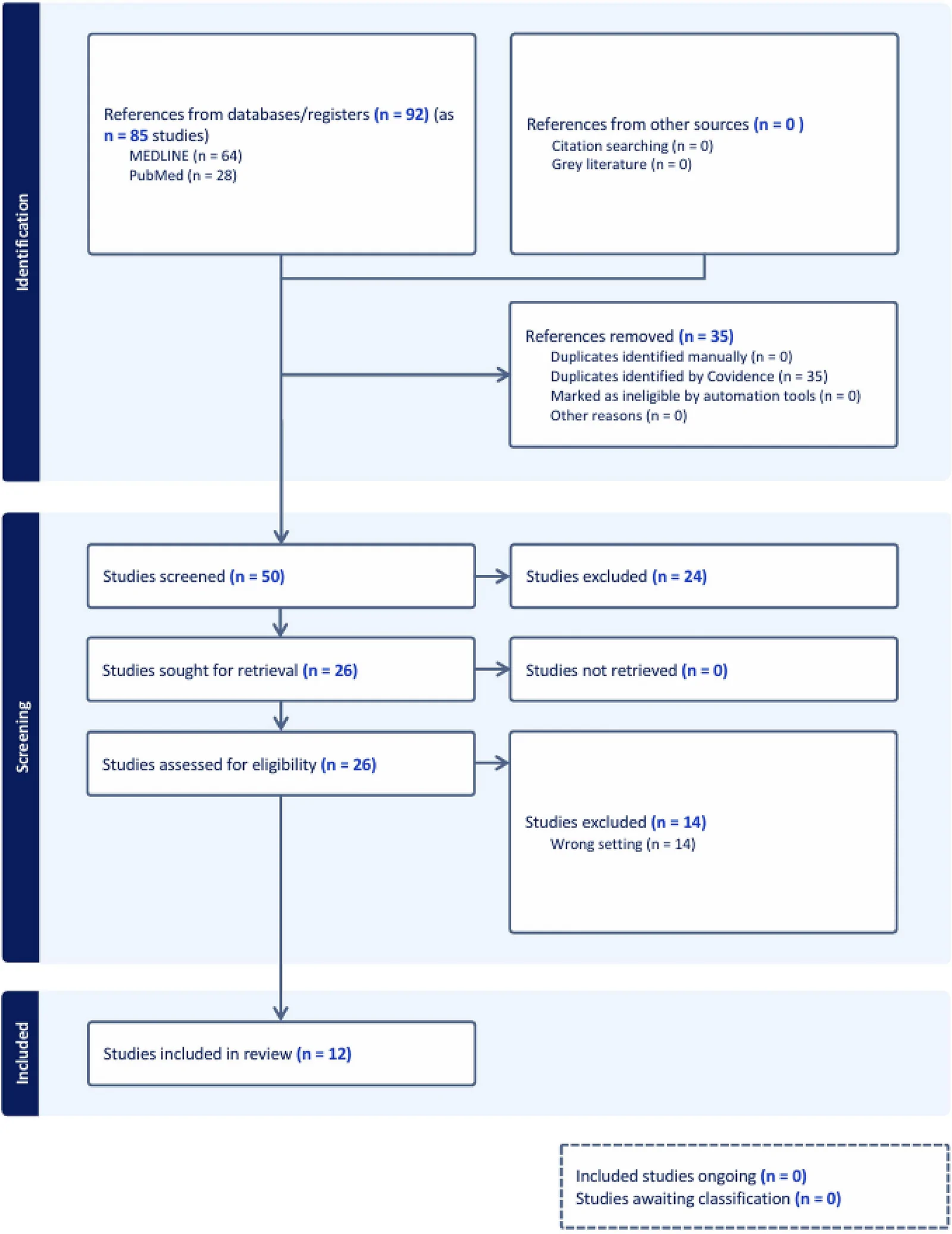
PRISMA chart

All identified records were imported into Covidence systematic review software (Veritas Health Innovation, Melbourne, Australia) for screening and management. Screening and study selection were performed independently by two reviewers following PRISMA guidelines. After removal of duplicates, titles and abstracts were screened for relevance. Full-text articles were then assessed to determine eligibility (Fig. 3).

Studies were included if they evaluated LESS in patients with adolescent idiopathic scoliosis and reported clinical outcomes related to curve progression, treatment effectiveness, or patient compliance. Studies involving animal models, non-idiopathic scoliosis populations, purely mechanistic experiments, or descriptive device reports without clinical outcomes were excluded. Disagreements between reviewers regarding study inclusion were resolved through discussion and consensus. Data from included studies were extracted in a structured format, including study design, patient population, curve magnitude, treatment protocol, follow-up duration, and reported outcomes.

### Data synthesis

Findings from the archival analysis and literature review were synthesized using a thematic historical approach, integrating chronological developments in LESS research with evolving clinical evidence. This approach allowed reconstruction of the lifecycle of LESS, from its early experimental development and clinical adoption to subsequent evaluation and eventual abandonment, while also situating the technology within the broader context of innovation in nonoperative scoliosis management.

## Results

### Study identification

Following PRISMA guidelines, archival materials from the Scoliosis Research Society (SRS) were systematically reviewed. A total of 24 abstract presentations related to lateral electrical surface stimulation (LESS) were identified within the SRS archives. These abstracts were independently reviewed by two investigators, and structured data including title, research institution location, study methods, primary results, and lessons learned were extracted.

In parallel, a focused literature review was conducted following PRISMA methodology. A total of 50 records were identified through searches of PubMed and Ovid MEDLINE. After screening by two independent reviewers, 26 articles underwent full-text review, of which 12 studies met the predefined inclusion criteria.

### ScoliTron™ overview

The ScoliTron™ is a portable electronic stimulator designed to deliver Lateral Electrical Surface Stimulation (LESS) therapy for the management of scoliosis [1]. The system applies electrical stimulation to the lateral trunk musculature through removable skin electrodes (Fig. 1), generating corrective forces on the spine. Developed from earlier neuromuscular electrical stimulation techniques used in rehabilitation, the device was intended as a noninvasive, home-based alternative to bracing or surgery.

The stimulator produces trains of rectangular constant-current pulses with a duration of 200 microseconds and a frequency of 25 pulses per second. Pulses are monophasic but capacitively coupled to prevent electrode polarization and reduce skin irritation. Current amplitude typically ranges from 60 to 80 mA, depending on patient characteristics such as subcutaneous fat thickness. The stimulation cycle mimics voluntary muscle contraction: current increases to the preset amplitude over 1.5 s, maintains contraction for 6 s, decreases to zero over 0.8 s, and is followed by a 6-s relaxation period, balancing corrective force application with avoidance of muscle fatigue. In clinical use, the device was typically worn 8 h during sleep, providing approximately 4 h of active stimulation per day without interfering with daytime activities.

Originally developed at the Rancho Los Amigos Rehabilitation Engineering Center (Downey, CA), the ScoliTron™ was developed in collaboration with Neuromedics, Inc. (Clute, TX). The single-channel unit included physician- and patient-controlled settings, rechargeable batteries, manual and automatic stimulation modes, and a built-in hour meter that recorded stimulation delivered above 40 mA to monitor treatment adherence. A dual-channel version was also available for patients with double curves. Electrical stimulation was delivered through carbon–rubber electrodes applied with conductive interface materials, including karaya pads, polymer pads, gel pads, and conductive gels, sometimes secured with medical tape to minimize skin irritation and maintain electrode contact. Together, these features enabled prolonged home-based muscle stimulation intended to apply corrective forces to the spine during growth.

### Temporal evolution of LESS research and clinical adoption

Analysis of SRS archival presentations demonstrated a clear trajectory in the development of LESS, characterized by early enthusiasm, rapid clinical adoption, and eventual decline. Early investigations established the biomechanical rationale for targeting lateral rather than paraspinal musculature. In 1977, Axelgaard et al. demonstrated that stimulation of the lateral trunk muscles produced greater spinal curvature than paraspinal stimulation, forming the basis for the therapeutic application of LESS [4].

These findings were supported by early experimental and clinical work suggesting that cyclic contraction of trunk musculature could generate corrective forces across the spine and potentially influence curve progression [3]. The concept of alternating stimulation of lateral muscle groups further suggested a mechanism for producing sustained corrective forces, contributing to growing clinical interest.

Initial clinical studies reported encouraging results. In 1979, Axelgaard et al. evaluated 33 patients, demonstrating curve improvement in five patients and stabilization in 21 at three months [2]. A subsequent 1981 study involving 107 patients reported stabilization or improvement in most cases, with high compliance and minimal skin complications [5]. These early findings suggested that LESS could provide a noninvasive, nighttime alternative to bracing, contributing to widespread adoption and multicenter clinical investigations.

### Clinical outcomes and treatment effectiveness

Across both archival abstracts and published studies, early investigations consistently suggested that LESS could produce short-term curve stabilization or modest improvement in selected patients [6, 7]. These outcomes were most frequently observed in patients with smaller curves and favorable biomechanical characteristics, with stimulation typically applied during nighttime using electrodes placed on the convex side of the curve [1].

In addition to biomechanical effects, studies also evaluated patient tolerance and psychosocial outcomes. Kahanovitz et al. reported that patients treated with electrical stimulation demonstrated fewer negative psychosocial responses compared with those treated with bracing [8]. Compliance studies further suggested that nighttime stimulation protocols were generally well tolerated, although adherence remained variable [9].

However, as larger cohorts were studied and follow-up periods increased, outcomes became increasingly inconsistent. In 1982, Bradford et al. reported that while 82% of patients with curves under 30° stabilized or improved, 72% of patients with curves over 30° progressed [10]. Similarly, multicenter studies by Brown et al. demonstrated modest stabilization but highlighted high dropout rates and compliance issues [11]. Throughout the mid-1980s, several additional clinical studies evaluated the effectiveness of electrical stimulation therapy. Investigations by Sullivan and colleagues and Bylund and collaborators reported variable clinical outcomes, with some patients demonstrating stabilization of curve progression and others exhibiting continued deformity progression despite treatment [12, 13]. These studies highlighted the importance of patient selection, curve magnitude, and skeletal maturity in determining treatment success.

Further investigations revealed significant limitations. Sullivan et al. reported high rates of treatment failure, particularly in skeletally immature patients during periods of rapid growth, with device malfunctions and unreliable compliance monitoring contributing to poor outcomes [14]. Studies focusing on patients at high risk of progression, including those analyzed using the Lonstein and Carlson criteria, demonstrated similarly unfavorable results, with no convincing evidence that LESS altered disease progression [15–17].

Comparative studies consistently demonstrated inferior or, at best, equivalent outcomes relative to bracing. One of the most significant comparative investigations was conducted by Durham and colleagues, who compared surface electrical stimulation with orthotic bracing in children with idiopathic scoliosis. Their findings suggested that bracing remained more effective at preventing curve progression, leading many clinicians to favor orthotic treatment over electrical stimulation [18]. Davis et al. reported comparable results only in highly selected patients with flexible curves, while broader populations showed significantly lower success rates [19]. Rowe et al. documented high dropout rates and device-related failures, with many patients transitioning to bracing [20]. Subsequent studies by O’Donnell et al. and Moskowitz et al. reported long-term failure rates exceeding 50% and consistently superior outcomes with bracing [21, 22]. These findings were further supported by later analyses, which confirmed that even among compliant patients, LESS did not meaningfully alter long-term curve progression [23].

Meta-analytic evidence reinforced these conclusions. Rowe et al. demonstrated that electrical stimulation provided limited benefit compared with brace treatment, which consistently showed superior effectiveness in preventing curve progression [24].

### Factors contributing to treatment failure

Across both archival and published studies, several factors consistently contributed to the limited effectiveness of LESS. These included reduced efficacy in patients with larger curves or significant growth potential, variable patient compliance with nightly treatment protocols, and technical limitations of the stimulation devices.

Device-related issues, including malfunction and unreliable compliance monitoring, were frequently reported and contributed to treatment discontinuation [14, 20]. Additionally, skin irritation and discomfort associated with prolonged electrode use affected patient tolerance. Although later experimental studies demonstrated that electrical stimulation could influence muscle morphology and neuromuscular function, the clinical relevance of these findings remained unclear [25].

## Discussion

The present study integrates SRS archival materials with published literature to reconstruct the full trajectory of lateral electrical surface stimulation (LESS), from early development and widespread clinical adoption to eventual abandonment. Across both data sources, a consistent pattern emerges: early enthusiasm driven by promising short-term findings was not sustained in larger cohorts with longer follow-up, and LESS ultimately failed to demonstrate durable clinical effectiveness.

A central finding of this analysis is that LESS did not alter the natural history of adolescent idiopathic scoliosis. While early studies suggested short-term curve stabilization in selected patients, subsequent investigations consistently demonstrated progression rates comparable to, or worse than, untreated or braced populations. This discrepancy highlights a critical limitation in the underlying conceptual model of LESS.

From a biomechanical perspective, the failure of LESS can be attributed to its inability to generate sufficient and sustained corrective forces to meaningfully influence spinal growth. Scoliosis is a complex three-dimensional deformity involving not only lateral curvature but also vertebral rotation and sagittal plane abnormalities [26]. Surface electrical stimulation, which primarily activates superficial trunk musculature, is inherently limited in its capacity to correct these structural components [27]. Although stimulation may transiently alter muscle activation patterns, the magnitude and duration of force generated are unlikely to overcome the biomechanical forces driving curve progression, particularly during periods of rapid skeletal growth.

In addition to biomechanical limitations, the biologic assumptions underlying LESS were overly simplistic. The premise that scoliosis progression is primarily driven by muscle imbalance does not account for the multifactorial nature of the condition, which includes asymmetric vertebral growth, genetic factors, and neuromuscular control mechanisms [28]. As a result, interventions targeting muscle activity alone are insufficient to modify the structural progression of the deformity [24]. Early favorable outcomes likely reflected short-term biomechanical effects, selection of lower-risk patients, and limited follow-up durations rather than true disease modification.

Comparative studies further reinforce these conclusions. Across multiple investigations, orthotic bracing consistently demonstrated superior effectiveness in preventing curve progression [24]. Unlike electrical stimulation, bracing applies continuous external forces that directly influence spinal alignment and growth modulation. These findings are consistent with modern treatment paradigms, in which both nonoperative and operative strategies are designed to address the structural and growth-related components of scoliosis. Contemporary approaches, including advanced three-dimensional bracing techniques and growth-modulation procedures, such as vertebral body tethering, reflect a more comprehensive understanding of the disease process and its biomechanical drivers [29, 30].

An important contribution of this study is the demonstration of how archival research can complement traditional literature review. Published manuscripts often represent only a portion of the scientific dialog surrounding a new technology. The SRS archives preserve preliminary reports, evolving clinical experiences, debates, and observations that may never reach formal publication. In the case of LESS, these materials reveal how enthusiasm developed, how concerns emerged, and how professional opinion gradually shifted as evidence accumulated. This broader perspective provides a more complete understanding of the innovation lifecycle than published studies alone.

This study has several limitations. The archival analysis relies heavily on meeting abstracts and historical materials, which frequently provide less methodological detail than peer-reviewed publications. Considerable heterogeneity existed among studies with respect to patient selection, curve magnitude, treatment protocols, and outcome measures, limiting direct comparison. Publication and reporting bias may also have influenced both the archival and published records, particularly during the early phases of adoption when positive findings were more likely to be disseminated. Additionally, the retrospective and descriptive nature of this analysis precludes quantitative synthesis beyond the available data. Nevertheless, the integration of archival and published evidence provides a unique longitudinal perspective that would not have been possible through either source alone.

The story of LESS extends beyond the evaluation of a single treatment modality. It serves as a case study in the complex relationship between innovation, evidence generation, clinical adoption, and eventual reassessment. As new technologies continue to emerge in spine surgery, the lessons from LESS remain highly relevant: promising concepts require rigorous validation, long-term follow-up, and reproducibility across institutions before widespread acceptance. Historical analyses such as this can help provide perspective and context as future innovations move through similar cycles of enthusiasm, evaluation, and refinement.

## Conclusion

The rise and fall of LESS represents more than the story of a treatment that ultimately failed to achieve its intended goals. It provides a valuable case study in how medical innovations emerge, gain acceptance, and are later reassessed as higher-quality evidence becomes available. Early enthusiasm for LESS was driven by promising preliminary results and the appeal of a non-invasive alternative to bracing. However, larger studies and longer follow-up demonstrated that the technology did not reliably alter the natural history of adolescent idiopathic scoliosis and was ultimately unable to justify widespread clinical use.

Equally important, this study highlights the value of historical archives in understanding the evolution of clinical practice. The SRS archives preserved observations, scientific presentation, discussions, and perspectives that extended beyond the published literature and helped reconstruct the full trajectory of LESS from innovation and adoption to decline and abandonment. As spine surgery continues to evolve, these lessons remain relevant. Meaningful innovation requires not only creativity and technological advancement, but also rigorous study design, thoughtful patient selection, reproducibility, and long-term evidence. History, when critically examined, remains one of the most powerful tools for guiding the future of patient care.

## Supplementary Information

Below is the link to the electronic supplementary material.

Supplementary file1 (DOCX 30 kb)

## Data Availability

Data is available upon request.
