# Supplementary material for: The rise and fall of lateral electrical surface stimulation (LESS) through the lens of SRS archives
Source: Spine Deform. 2026 Jul 27;14(5):1519–27. doi: 10.1007/s43390-026-01510-y (PMC13553607; doi:10.1007/s43390-026-01510-y)
Supplement: Supplementary file 1 [file 43390_2026_1510_MOESM1_ESM.docx]

**Supplement Timeline**

**Table 1** Timeline of the abstracts related to LESS presented at Scoliosis Research Society Annual Meetings

| Item # | References | Title | Research institute location | Methods | Primary results | Lessons learned |
| --- | --- | --- | --- | --- | --- | --- |
| 1 | Axelgaard et al., 1977 | Lateral Surface Stimulation for the Correction of Scoliosis | Downey, CA | This study examines whether lateral trunk muscle stimulation improves curvature in paralytic scoliosis versus traditional paraspinal stimulation. Thirty-three idiopathic scoliosis patients were screened for optimal electrode placement, and those with positive responses received overnight Lateral Electrical Surface Stimulation (LESS). | Screening process revealed that lateral trunk muscle stimulation was more effective than paraspinal stimulation in reducing curvature of the spine. The alternating stimulation method (switching between lateral muscle groups) provided continuous corrective forces, unlike traditional on-off methods. | Targeting lateral muscles offers biomechanical advantages in scoliosis correction. |
| 2 | Axelgaard et al., 1979 | Transcutaneous Electrical Muscle Stimulation for the Treatment of Idiopathic Scoliosis-Preliminary Results | Downey, CA | This study evaluates transcutaneous lateral electrical muscle stimulation for treating idiopathic scoliosis in growing juvenile and adolescent patients. Thirty-three participants were screened to identify optimal electrode placement, and those with positive responses subsequently underwent overnight Lateral Electrical Surface Stimulation (LESS) with follow-up evaluations at 3, 6, and 9 months. | **Three-month follow-up (26 patients):** 5 showed improvement (≥5° correction). 21 had arrest of progression (≤+5° change). **Six-month follow-up:** 3 improved. 11 showed arrest of progression. 1 worsened. **Nine-month follow-up (8 patients):** 1 improved. 7 showed arrest of progression. **Key observation:** Pre-treatment curve progression was 10° per six months, but after LESS treatment, only one of 26 patients showed continued progression. | LESS therapy shows potential effectiveness in halting scoliosis progression in growing patients. Stimulation of lateral trunk muscles was more effective than paraspinal muscle stimulation. The therapy is non-invasive and applied during sleep, making it a promising alternative for scoliosis management. |
| 3 | Axelgaard et al., 1981 | Kyphosis Treatment by Electrical Surface Stimulation | Downey, CA | This study evaluates neuromuscular electrical surface stimulation as a non-bracing treatment alternative for Scheuermann’s kyphosis and postural roundback. Eight patients with progressive Scheuermann’s kyphosis received nighttime paraspinal muscle stimulation with electrode placement tailored to curvature type, and treatment effectiveness was assessed over 1–1.5 years of follow-up. | After 1 year: Average kyphosis reduced from 51° to 46°. After 1.5 years: Further reduction to 42°. All patients showed kyphosis correction, with either reduced or unchanged vertebral wedging. One patient who improved by 13° over 27 months maintained the correction 1-year post-treatment at skeletal maturity. | Electrical stimulation is a feasible alternative to bracing, with potential benefits in kyphosis correction. Side effects appear minimal, and patient compliance is better than with bracing. |
| 4 | Axelgaard et al., 1981 | Scoliosis Treatment by Lateral Electrical Surface Stimulation | Downey, CA | This study evaluates Lateral Electrical Surface Stimulation (LESS) as a non-operative alternative to bracing for progressive scoliosis in patients with 20–45° curves and radiographic progression. Nighttime stimulation targeted lateral trunk muscles, with follow-up extending up to 4 years of treatment and 1 year beyond skeletal maturity. The cohort comprised 107 patients: 65 with single major idiopathic curves, 9 with double major idiopathic curves, 10 with neuromuscular scoliosis, and 3 with miscellaneous scoliosis. | Idiopathic scoliosis group:  ◾Pre-treatment progression rate: 2.1° per month  ◾During treatment: Average curvature reduction of 2°  ◾Standard deviation of curvature results in the first 2 years: 3°  Post-treatment (3 patients): No progression observed.  Rib hump reduction (measured in 13 patients after 2 years): 2 mm  Overall patient compliance: 90%  Treatment success (arresting or improving curvature): 96%  Complications: Minor skin rash, but no patient had to discontinue treatment in the last 2 years due to persistent irritation. | LESS is a feasible and effective alternative to bracing for progressive scoliosis. High patient compliance (90%) suggests it is better tolerated than bracing. No known side effects aside from minor skin irritation, which has been resolved with improved materials. |
| 5 | Bradford et al., 1982 | Surface Electrical Stimulation in the Treatment of Idiopathic Scoliosis: Preliminary Results in 29 Patients | Minneapolis, MN | This study evaluates surface electrical stimulation for idiopathic scoliosis in skeletally immature patients. Of 29 patients followed for ≥1 year, five were excluded for non-compliance or skin intolerance, leaving 24 who wore an Electrical Stimulating Orthosis for ≥8 h daily with radiographically guided electrode placement, most commonly at the midscapular position (19/24). All but two patients had thoracic curves; two had juvenile idiopathic scoliosis, and the remainder had adolescent idiopathic scoliosis. | 1 patient improved. 11 patients stabilized. 4 had minor progression (progression less than 10  degrees to a curvature of 35 degress or less not requiring further treatment). 8 patients had significant progression, requiring further treatment: 2 needed surgery. 6 switched to a Milwaukee brace, but 4 of them eventually required surgery due to continued progression. Outcomes by curve severity at treatment start: Curves <30°: 82% stabilized or improved. Curves >30°: Only 28% stabilized, while 72% progressed. 8 patients were still undergoing active treatment at the time of reporting. | Electrical stimulation may be effective in some patients, particularly those with curvatures <30°. Patients with curves >30° are less likely to benefit and more likely to require surgery. If electrical stimulation fails to control progression, a Milwaukee brace is unlikely to be effective either. |
| 6 | Brown et al., 1982 | Multicenter Trial of a Noninvasive Stimulation Method for Idiopathic Scoliosis | Houston, TX | This study evaluates Lateral Electrical Stimulation (LES) as a bracing alternative for scoliosis in routine clinical practice. A total of 548 patients (484 females, 64 males; age 5.1–18.6 years, mean ≈12.9) were treated across 58 sites, including 100 high-risk skeletally immature patients with 20–45° curves and 448 general patients. Treatment lasted up to 50.9 months with follow-up to 12 months post-treatment; 51 patients (30%) dropped out, and 26 (15%) experienced curve progression >5° (mean 9.6°). | 28 patients completed treatment and reached skeletal maturity.  4 showed >5° progression (range: 6–10°; mean: 7.5°).  Overall curve changes ranged from −8° (improvement) to +10° (progression), with a mean change of 0.3°.  No serious adverse effects.  Most common side effect: Skin irritation from adhesive electrodes, successfully managed in all but 3 cases.  No specific risk factors predicted treatment dropout. | Lateral electrical stimulation appears to stabilize actively progressing scoliosis. Well-tolerated and feasible as an alternative to bracing. Effective for curves with an apex at T5 or below. |
| 7 | Frontino et al., 1982 | The Surface Electrical Stimulation (L.E.S.S.) for the Treatment of Idiopathic Progressive Scoliosis | Florence, Italy | This multicenter study investigates Lateral Electrical Surface Stimulation (LESS.) as a non-invasive treatment for progressive idiopathic scoliosis. Between March 1979 and March 1982, 182 patients were treated, including 80 with single major curves (44%) managed with single-channel stimulation and 20 with double major curves (11%) managed with dual-channel stimulation; follow-up ranged from 9 to 36 months, while 82 patients (45%) were excluded due to follow-up shorter than 9 months. | Both groups showed curve stabilization within 5° tolerance. 25% of thoracolumbar curves improved by an average of 31.5%. Rib hump reduction: 50% in both groups. Treatment effectiveness: 85% of considered patients had scoliosis progression arrested. 11% (11 patients) showed ineffective results. 4% (4 patients) withdrew due to intolerance. | Preliminary but encouraging results for fast-progressive scoliosis cases. Dual-channel stimulation is more effective for double major curves. Further research needed for long-term outcomes and optimization of device practicality. |
| 8 | Axelgaard et al., 1983 | Spinal Biomechanics of Surface Muscle Stimulation | Downey, CA | This study identifies optimal surface electrode placement for non-surgical correction of spinal deformities in skeletally immature patients, including scoliosis, kyphosis, lordosis, and combined patterns, using neuromuscular electrical stimulation (70 mA, 200 µs, 30 Hz) with recumbent radiographic assessment. The cohort included 32 scoliosis patients with 35 major curves (mean 25°), 18 kyphosis patients (mean 57°), and one lordosis patient (88°). | Scoliosis: Lateral electrode placement provided the highest correction (45%), followed by intermediate (38%) and medial (16%). Axial rotation was reduced by ~4° in all positions. Kyphosis: Reduced by 28% (from 57° to 34°) with medial electrode placement. Lordosis: Reduced from 88° to 48° with stimulation of the rectus abdominis. | Lateral electrode placement is optimal for scoliosis correction. Medial placement on the paraspinals is best for kyphosis. Rectus abdominis stimulation is effective for lordosis reduction. Curve correction is strongly related to the lever arm length connecting the stimulated muscles to the spine. |
| 9 | Kahanovitz et al., 1983 | The Comparative Results of Prospective Psychological Testing in Idiopathic Scoliosis Patients Treated with Electrical Stimulation or Bracing | New York, NY | This study prospectively compares Lateral Electrical Surface Stimulation (LESS) with TLSO or Milwaukee bracing in idiopathic scoliosis to assess psychological outcomes using seven standardized psychological tests and observer ratings by physicians or physical therapists. All participants were female, although specific ages and curve severities were not reported. | Self-esteem: LESS patients had significantly higher self-esteem than brace patients. Coping Mechanisms: Brace patients showed more avoidance-based coping, whereas LESS patients coped more actively. Mood & Activity: LESS patients were more physically active, outgoing, and vigorous, while brace patients were more withdrawn.  Emotional State: Brace patients exhibited more anxiety, depression, helplessness, and discomfort compared to LESS patients. | LESS treatment provides notable psychological benefits over bracing.  Patients in the LESS group had better coping strategies, lower emotional distress, and higher engagement in treatment.  The study supports LESS as a preferable alternative to bracing in terms of psychological well-being. |
| 10 | Sendele et al., 1983 | In Vivo Three Dimensional Roentgenographic Evaluation of Surface Electrical Stimulation in Scoliosis | Cleveland, OH | This study evaluates whether lateral surface electrode muscle stimulation reduces scoliosis curvature, vertebral rotation, and sagittal deformity in adolescent idiopathic scoliosis. Seven female patients with single (*n* = 6) or double (*n* = 1) major curves (eight curves total; 23°–41°, mean 31°) underwent 3D radiographic analysis using erect, prone, and prone-with-stimulation X-rays acquired in a biplane frame to model vertebral positions. | Curve Correction: Prone positioning alone reduced scoliosis by 31.5% (to 21.25°). Adding muscle stimulation further reduced curvature by 31.5% (to 11.5°), achieving a total correction of 63%. Kyphosis/Lordosis: Patients had pre-existing thoracic hypokyphosis (mean 6.67°). Prone positioning led to mild lordosis (−0.33°), with minimal additional lordosis (−1.67°) from stimulation. Vertebral Rotation: Maximum vertebral rotation was 7.42° in scoliosis patients (compared to ±2° in non-scoliotics). Prone positioning caused minor derotation, but stimulation did not produce significant additional vertebral derotation. | Lateral surface muscle stimulation in the prone position significantly reduces scoliosis curvature, but half of the correction is due to the prone posture itself. Stimulation does not significantly improve vertebral derotation. Thoracic hypokyphosis is mildly worsened by prone positioning and stimulation. |
| 11 | Sullivan et al., 1983 | Observations on the Use of the Scolitron in the Management of Scoliosis | Oklahoma City, OK | This study reports preliminary outcomes of the ScoliTron for idiopathic scoliosis based on a retrospective review of 26 patients from two centers, including assessments of curve progression, treatment duration, compliance, complications, and reasons for treatment failure. The cohort comprised patients with idiopathic scoliosis (mean age 12 years, range 9–15; mean initial curve 28°, range 17–40°), with some referrals occurring due to refusal to wear a brace, introducing potential compliance bias. | **Failure Rate**: 16 patients (61.5%) were classified as treatment failures, with an average curve progression of 10°.  ◾13 transitioned to bracing.  ◾1 required surgery after 13 months.  ◾2 patients progressed despite not using the device.  **Compliance Issues**: 6 patients were non-compliant, including 2 who did not progress despite no treatment.  ◾ 4 of the 16 failures were non-compliant.  **Mechanical Issues**: Device failures included battery problems, coupling defects, and an ineffective compliance meter.  **Complications**: Over half of the patients experienced skin irritation.  Skeletally Immature Patients: Long-term use (1–2 years pre-puberty) was ineffective, with progression occurring during rapid growth. | ScoliTron was less effective than expected, particularly in non-compliant or skeletally immature patients.  Mechanical and supply issues contributed to frustration and treatment challenges.  Double major curves were difficult to manage with the device.  A refined unit with improved compliance tracking and dual-channel stimulation may improve future outcomes.  Caution is advised in adopting this method widely. |
| 12 | Adler et al., 1984 | Management of Skin Irritation During Electrical Stimulation for Scoliosis | Palo Alto, CA | This study investigates the causes, prevention, and management of skin irritation from electrical stimulation in idiopathic scoliosis. A series of 36 patients undergoing stimulation was analyzed for irritation causes and management strategies. | Incidence of Skin Irritation: Reported in 20% of patients (compared to previously reported 50%). The abstract also mentioned several causes and prevention strategies for skin irritation due to LESS, which are irrelevant to our study | With appropriate interventions, skin irritation does not have to be a limiting factor for electrical stimulation therapy. |
| 13 | Brown et al., 1984 | Impact of L.E.S.S.Treatment on Natural History of Patients at High Risk of Curve Progression | Downey, CA | This study retrospectively evaluates whether Lateral Electrical Surface Stimulation (LESS) halts curve progression in high-risk adolescent idiopathic scoliosis compared with the expected natural history. The ISO-LESS multicenter database included 132 female patients treated for ≥1 year who were skeletally immature (bone age ≤13 years), pre-menarchic, Risser 0–1, and met curve criteria of 30–45°, 20–24° with ≥10° progression within 6 months, or 25–29° with ≥5° progression within 3 months. | **Curve Magnitude** (Mean ± SD): 32.8° ± 2.6. **Skeletal Age** (Mean ± SD): 11.3 ± 1.6 years. **Progression During Treatment**: 46 patients (35%) progressed ≥6° at some point during treatment (includes non-compliant patients). Favorable comparison to previously reported natural history probabilities: Natural history data suggested a 48–90% probability of progression in untreated high-risk patients. **Progression Velocity:** Pre-treatment: 1.82°/month. During LESS treatment: -0.07°/month (indicating curve stabilization or regression). **Interpretation**: LESS stimulation significantly reduced curve progression rates. Treatment successfully halted progression at the peak risk period for worsening scoliosis. | LESS stimulation is highly effective in stopping scoliosis curve progression in high-risk patients. Results suggest better outcomes compared to untreated natural history progression rates. Patient compliance remains a key factor in treatment success. |
| 14 | Whiffen et al., 1984 | Treatment of Scoliosis in the Severely Retarded Cerebral Palsy Patient by Electrical Surface Stimulation—a Preliminary Report | Madison, Wl | This observational study evaluates whether electrical surface stimulation can arrest or reduce scoliosis progression in patients with spastic cerebral palsy and severe developmental delay until spinal maturity allows surgery. Nine patients with curves >35° and substantial remaining growth were enrolled, eight of whom were followed for at least 5 months over a 2-year observation period. | **Curve Reduction**: 4 patients saw a decrease in curve size (average initial curve 70.5°, reduced by an average of 11°).  **Stabilization**: 2 patients had no progression.  **Progression**: 2 patients experienced curve worsening.  Both were outpatients.  Failure was attributed to mechanical device issues and skin rashes.  **Intervening Factors**:  One patient experienced an 18° curve deterioration when treatment was paused due to seizures, but reapplication led to a 5° improvement.  Another patient had initial curve reductions (from 66°/71° to 54°/64°) but regressed during a two-month treatment hiatus due to gastrointestinal surgery.  **Electrode Placement Strategy**:  Initial placement in the paraspinal region for 3–6 months before transitioning to a midaxillary position. | Electrical stimulation is a feasible treatment option for scoliosis in patients with cerebral palsy. |
| 15 | Akbarnia et al., 1985 | Lateral Electrical Surface Stimulation (LESS) for the Treatment of Adolescent Idiopathic Scoliosis (A.I.S.). an Analysis Based on Progression Risk | St. Louis, MO | This study retrospectively evaluates whether Lateral Electrical Surface Stimulation (LESS) alters the natural history of adolescent idiopathic scoliosis in patients at risk for curve progression. Twenty-two patients treated since July 1982 at Cardinal Glennon Children’s Hospital were reviewed; six were excluded (two juvenile cases, three with curves >30°, and one dropout), leaving 16 adolescents with curves of 20°–30° for analysis. | Average initial curve: 28.5°. Supine correction: 16.5°, further reduced to 8.9° with stimulation. Average stimulation: 67 mA. Compliance issues in 25% of patients. **Failure Rate:** Overall: 7/16 patients (43%) progressed by >5° (average progression 10.6°). Among high-risk patients (n=11): 6/11 (54%) progressed despite LESS. **Compliance impact**: 2/6 failures were non-compliant. 5 compliant patients did not progress. **Predicted vs. Observed Progression**: Logistic regression predicted 7 patients would progress. 4/7 (57%) did progress despite treatment. Only 1 patient showed potential alteration of natural history due to LESS. | LESS did not consistently alter scoliosis progression in high-risk patients. The treatment had a high failure rate (43–54%), even among compliant patients. The study supports maintaining LESS as an experimental treatment, requiring further research in specialized centers with larger patient cohorts. |
| 16 | Brown et al., 1985 | Multicenter Study of 1221 Patients on Lateral Electrical Surface Stimulation for Idiopathic Scoliosis | Downey, CA | This longitudinal study assesses Lateral Electrical Surface Stimulation (LESS) for halting idiopathic scoliosis progression in 1,221 patients (137 males, 1,076 females) treated with single-channel LESS from 1977 to 1985. Mean initial curve was 28°, with 90% Risser ≤2. Mean skeletal age was 12.9 years in males (83% ≤14) and 12.6 years in females (77% ≤13), and mean age at treatment was 13.5 and 12.9 years, respectively. Pre-treatment progression averaged 1.2°/month in the 3–6 months before therapy, and outcomes were followed up to 3-year post-treatment. | **Overall Effectiveness**:  Progression halted after initiating stimulation. Pre-treatment curve increase: 7.2% over 6 months. During treatment: Progression reduced to 0.2° on average.  **Curve Type-Specific Outcomes**:  Thoracic curves: +0.8° (slight increase). Thoracolumbar curves: -1.5° (decreased). Lumbar curves: -0.3° (slight decrease).  **Long-Term Results (Post-Treatment):**  Change of only 0.3° up to 3 years after treatment ended.  **Patient** **Outcomes**:  52% still in treatment. 19% reached skeletal maturity. 10% progressed despite LESS.  19% discontinued for other reasons.  **Overall Success Rate: 83%.** | LESS appears effective in halting or slowing curve progression, especially for curves under 45°.  Best results observed in thoracolumbar and lumbar curves compared to thoracic curves.  Long-term stability was observed post-treatment, with minimal curve change up to 3 years.  A small subset (10%) still progressed despite LESS, highlighting the need for individualized risk assessment. |
| 17 | Sullivan et al., 1985 | Further Evaluation of the ScoliTron® Treatment of Idiopathic Scoliosis | Oklahoma City, OK | This retrospective multicenter study evaluates whether Lateral Electrical Surface Stimulation (LESS) alters the natural history of idiopathic scoliosis compared with bracing in high-risk patients. A total of 144 patients were reviewed across five centers, including 48 protocol, 60 non-protocol, and 36 undefined cases, with a mean initial curve of 28°, and outcomes assessed included curve progression, complications, and overall treatment results. | At the last follow-up, the average curve was 32°; 65% (79 patients) experienced failure (≥10° progression), while 35% (42 patients) were considered successful. Over half of the successful cases were non-protocol patients, raising concerns about treatment effectiveness. | It cannot be said that the natural history of idiopathic scoliosis has been altered by lateral electrical surface stimulation. Until sufficient numbers of protocol patients are treated and followed past maturity, the authors cannot be certain that this method is as effective as brace wear. |
| 18 | Davis et al., 1986 | Efficacy of Electrical Surface Stimulation Versus Bracing in the Treatment of Idiopathic Scoliosis—a Comparative Study | Columbia, SC | This retrospective study evaluates whether external surface stimulation (ESS) is as effective as bracing for idiopathic scoliosis and identifies responsive subgroups by comparing curve progression among patients meeting ESS criteria (25°–35° curves, ≥50% side-bending flexibility, normal or increased kyphosis, Risser ≤2, vertebral rotation ≤12.5°) and non-criteria groups. The cohort included 27 criteria ESS patients, 33 criteria bracing patients, 38 non-criteria ESS patients, and 52 non-criteria bracing patients. | Criteria Groups: ESS had an 85% success rate (mean curve change: 27.8° → 27.0°), while bracing had 82% success (28.7° → 24.6°). Noncriteria Groups: ESS had only 23% success (31° → 29.8°), whereas bracing was more effective at 65% success (34.5° → 30.5°). Failure rates were higher in noncriteria groups, with ESS at 77% failure (32.3° → 44.3°) and bracing at 35% failure (34.7° → 43.6°). | ESS can be an effective alternative for select patients with flexible curves (25–35 degrees) and specific skeletal characteristics. However, for patients outside these criteria, bracing remains the more effective treatment option. |
| 19 | Rowe et al., 1986 | Comparison of Patient Acceptance of ScoliTron and Brace Treatment of Idiopathic Scoliosis | Kalamazoo, Ml | This retrospective study evaluates whether transcutaneous muscle stimulation (Scolitron) improves compliance compared with bracing in idiopathic scoliosis among high-risk patients meeting Scolitron entrance criteria. The Scolitron group included 15 patients (20 treated curves; mean age 12.25 years; mean Risser 0.40; mean initial curve 24.63°, final 31.40°), while the bracing group comprised 13 patients (16 treated curves; mean age 12.42 years; mean Risser 0.76; mean initial curve 23.24°, final 31.53°). | **Scolitron Group**: Only 1 curve successfully completed treatment, while 18/20 (90%) required a change in treatment or dropped out. Dropout rates were 6/20 at 6 months, 12/20 at 12 months, and 19/20 at 30 months. Two cases required surgery.  **Brace Group**: Higher compliance, with 100% retention at 12 months and only 3/16 dropping out by 48 months. One patient required surgery at 18 months, another at 48 months. | Scolitron treatment had poor compliance due to patient discomfort, painful skin lesions, and equipment failure. Patients who switched to bracing reported greater satisfaction despite initial reluctance. The study does not support claims that Scolitron is better accepted than bracing. |
| 20 | Carr et al., 1987 | Electrical Stimulation for Treatment of Idiopathic Scoliosis in a Group of High Risk Progressive Patients | Portland, OR | This retrospective study evaluates whether transcutaneous muscle stimulation (Scolitron) improves compliance compared with bracing in idiopathic scoliosis among high-risk patients meeting Scolitron entrance criteria. Patients were stratified by initial curve magnitude: <25° (7 patients, 8 curves; mean age 13.2 years; mean treatment duration 1.9 years), 25–29° (19 patients, 20 curves; mean age 12.6 years; mean treatment duration 1.2 years), and ≥30° (33 patients, 34 curves; mean age 12.8 years; mean treatment duration 1.3 years). | **Curves <25°:** 4 improved, 4 worsened, 1 required bracing. **Curves 25–29°:** 7 improved, 1 remained stable, 12 worsened, 9 required bracing, 6 required surgery. **Curves ≥30°:** 7 improved, 2 remained stable, 25 worsened, 4 required bracing, and 7 ultimately required surgery. | Electrical stimulation may help in small, early-stage curves but is ineffective for curves >25°, with 25% of these patients ultimately requiring surgery. The author recommends limiting its use to curves <30° that show early positive response; otherwise, brace treatment should be initiated if progression of 5° occurs. |
| 21 | O'Donnell et al., 1987 | Electrical Stimulation in the Treatment of Idiopathic Scoliosis | Wilmington. DE | This retrospective study evaluates the effectiveness of electrical stimulation for adolescent idiopathic scoliosis and compares outcomes with bracing and natural history in 172 patients treated since 1982 who had 20°–40° curves, Risser 0–2, and no prior treatment, with assessments of compliance, curve progression, and need for surgery. Among 118 protocol patients, 74 completed or failed treatment, while 44 remained in treatment; the mean patient age was 12.5 years, and over 80% were Risser 0 at treatment initiation. | **Patients still in treatment (44):** Average curve increase of 1.2°, 11.5% failure rate (curves >40°). **Completed treatment (56 fully compliant patients):** 66% had pretreatment curves of 20°–29°. Thoracic curves worsened by >10° on average. 33% of thoracolumbar/lumbar curves worsened by >10°. 20% required spinal fusion. 29% had curves >40° at follow-up. | While early results suggested failure rates similar to bracing, long-term analysis revealed a ~50% failure rate (curves >40° or requiring fusion). Electrical stimulation does not appear to improve outcomes compared to the natural history of adolescent idiopathic scoliosis. |
| 22 | Missiuna et al., 1989 | Electrical Spinal Instrumentation (E.S.I.): the Long Term Results | Toronto, ON | This retrospective study evaluates whether Electrical Spinal Instrumentation (ESI.) alters the natural progression of adolescent idiopathic scoliosis compared with untreated patients. A total of 318 patients with initial curves >20° were followed to skeletal maturity or ongoing treatment, assessing curve progression, skeletal maturity, and demographics; 210 patients met protocol criteria, with subgroup analyses by curve location (thoracic, lumbar, thoracolumbar, double) and magnitude (20–29°, 30–39°, ≥40°). | Curve progression (≥5° increase) mirrored that observed in untreated patients from natural history studies.  Detailed subgroup results are pending, but suggest no significant impact of ESI. on altering scoliosis progression. | ESI. does not appear to improve outcomes compared to the expected natural history of adolescent idiopathic scoliosis. These findings question the long-term effectiveness of ESI. as a non-operative treatment alternative. |
| 23 | Moskowitz et al., 1989 | ScoliTron Vs. Brace in Treatment of Idiopathic Scoliosis | Albany, NY | This retrospective study evaluates whether the Scolitron electrical stimulation device prevents curve progression in idiopathic scoliosis compared with bracing in skeletally immature patients (Risser 0–1) with documented progression. Of 40 treated patients, 32 patients (34 curves; mean age 12.8 years) were analyzed, with outcomes classified as successful, successful/failure, or failure after a mean treatment duration of 31 months. | **Failures**: 13 patients (45%) after 15.4 months of treatment. **Successful/Failure**: 6 patients (21%). **Successful**: 10 patients (34%) after 24.9 months of treatment, followed for 19.4 months post-maturity. No pretreatment factors (plumbline, rotational prominence, radiographic parameters) reliably predicted success. More flexible curves showed a trend toward better outcomes but were not statistically significant. **Bracing success rate**: 78%. | The Scolitron is not recommended for treating progressive idiopathic scoliosis, as its success rate is significantly lower than bracing. |
| 24 | Tiefel et al., 1989 | The Significance of Compliance in the Treatment of Idiopathic Scoliosis Using Electrical Surface Stimulation | Lakewood, CO | This study evaluates whether compliance with electrical stimulation influences outcomes in adolescent and juvenile scoliosis. Lakewood Orthopedic Clinic reviewed 130 patients treated since 1979 (80 responded), classifying them as compliant (90–100% usage) or noncompliant, with Cobb angles 20–40°, documented progression ≥5° for 20–29° curves, and ≥1 year of remaining bone growth. | Original protocol (80 patients):  Compliant group (47 patients): 57% avoided surgery, 43% required surgery. Noncompliant group (33 patients): 52% avoided surgery, 48% required surgery. Stricter protocol (Davis Criteria, 33 patients): Compliant group (19 patients): 74% avoided surgery, 26% required surgery. Noncompliant group (14 patients): 71% avoided surgery, 29% required surgery. | Compliance with electrical stimulation did not significantly impact outcomes in juvenile and adolescent scoliosis patients. These findings support Dr. J. Andy Sullivan’s argument that electrical stimulation does not alter the natural history of scoliosis. |
